# Very early orthodontic treatment: when, why and how?

**DOI:** 10.1590/2177-6709.27.2.e22spe2

**Published:** 2022-06-10

**Authors:** Ute E. M. SCHNEIDER-MOSER, Lorenz MOSER

**Affiliations:** 1 Private practice (Bolzano, Italy).; 2 University of Ferrara, Ferrara School of Orthodontics (Ferrara, Italy).; 3 University of Pennsylvania, School of Dental Medicine (Philadelphia/PA, USA).

**Keywords:** Interceptive orthodontics, Deciduous dentition, Simple mechanics, Serial extractions

## Abstract

**Introduction:**

Several orthodontic problems should already be treated at an early age to prevent the necessity of future complex and expensive procedures. Scientific evidence suggests that posterior crossbites, mild to moderate Class III, as well as certain Class II malocclusions, open bites and arch length discrepancies can benefit from simple, but efficient interceptive therapy.

**Objective:**

To summarize the existing evidence-based literature on early orthodontic treatment, and to illustrate its application and effectiveness by showcasing multiple clinical examples.

**Conclusion:**

Early short-term interceptive orthodontic treatment with simple appliances, in the deciduous or early mixed dentition phase, can efficiently correct certain malocclusions and help to either reduce the complexity or even avoid the necessity of complex and expensive procedures during puberty. For certain patients with significant arch length discrepancy the concept of serial extractions should be part of the orthodontic armamentarium.

## RESUMO

**Introdução:**

Vários problemas ortodônticos devem ser tratados já em idade precoce, para evitar a necessidade de procedimentos futuros de maior complexidade e custo. A evidência científica sugere que as seguintes más oclusões podem se beneficiar de terapias interceptivas simples mas eficientes: mordidas cruzadas posteriores, Classe III leve a moderada, certas más oclusões de Classe II, mordidas abertas e discrepâncias no tamanho das arcadas.

**Objetivo:**

Resumir a evidência científica existente sobre o tratamento ortodôntico precoce e ilustrar sua aplicação e efetividade, por meio da exposição de múltiplos casos clínicos.

**Conclusão:**

O tratamento ortodôntico interceptivo precoce de curto prazo com aparelhos simples na fase da dentição decídua ou início da dentição mista pode corrigir eficientemente certas más oclusões e ajudar a reduzir a complexidade ou, até mesmo, evitar a necessidade de procedimentos complexos e onerosos durante a puberdade. Para certos pacientes com discrepância significativa no comprimento das arcadas, o conceito de extrações seriadas deve fazer parte do arsenal de recursos ortodônticos.

## INTRODUCTION

The American Association of Orthodontists recommends that children should get their first check-up with an orthodontic specialist at the first recognition of a developing orthodontic problem, but no later than 7 years of age. Research has shown that certain malocclusions can benefit from early intervention and can help to either reduce the duration or even avoid the necessity of a more substantial and more expensive treatment at a later stage - not to mention the positive effect on the child’s quality of life by resolving psychosocial problems related to the malocclusion, as pointed out by Artese^1^ in 2019. 

In the case of uni- or bilateral posterior crossbites and Class III malocclusion, enough evidence-based literature is available to proof that a relatively short phase of interceptive treatment with simple appliances can normalize anomalous growth, and that the result of this treatment approach will remain stable over time. On the other hand, the existing literature on the benefits of early intervention for Class II, open bite and significant arch length discrepancy is controversial, which means that the clinician often must rely on her or his previous orthodontic education and acquired clinical experience. 

The possible advantages of the early intervention are the emotional satisfaction of the child, the growth potential available at this stage of development, greater collaboration with treatment, the possibility of a more simplified second phase, and the possible reduction of extractions in the corrective phase of treatment.

Thus, the aim of the present article is to summarize the current state of the art on early, or very early, orthodontic treatment, to present the evidence-based literature on the topic and, for situations where research is controversial, to provide the readers with simple short-term treatment approaches that proved to be efficient in the vast experience of the authors.

## POSTERIOR CROSSBITE

Posterior crossbites in the deciduous dentition are frequent findings, with a reported prevalence of 8-22%.^2^ ^,^ ^3^ The origin of these crossbites is a constriction of the maxilla, with an associated maxillary arch length discrepancy, which can lead to functional mandibular shifts caused by tooth interferences. Roughly 80% of all unilateral posterior crossbites in the mixed dentition are due to these functional shifts and, although spontaneous correction has been reported, it is more likely that the crossbite will be transferred to the permanent dentition and will cause asymmetrical muscle activity and mandibular growth, with an increased risk for future temporomandibular joint dysfunction.^4^ ^-^ ^15^

For preventing these negative looming sequelae, early orthodontic intervention is advisable as soon as the patient and the parents accept treatment, for normalizing the occlusion, with subsequent normal occlusal development by preventing the first permanent molars to erupt in crossbite, and to avoid future longer and more complex orthodontic treatments.^16^ ^-^ ^21^

**Figure 1:**
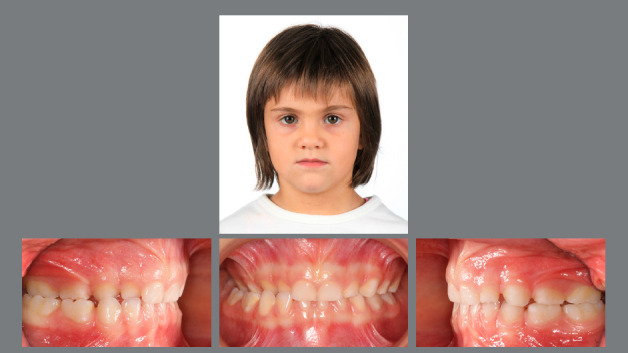
Pretreatment photographs at 5 years of age evidence a posterior crossbite on the right side.

**Figure 2:**
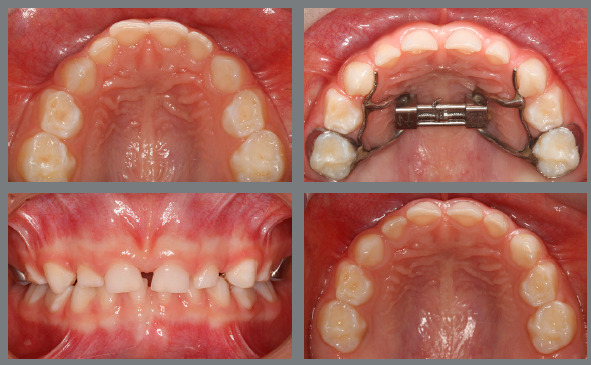
Before, during and after rapid palatal expansion (RPE).

**Figure 3:**
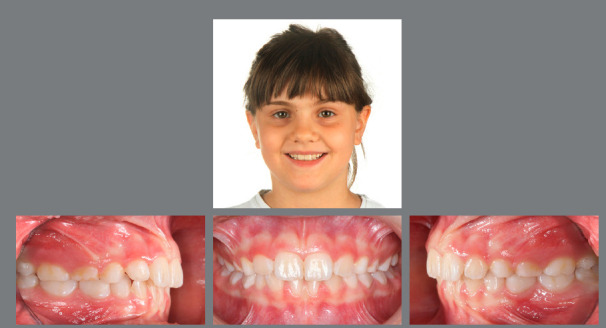
Good stability during the mixed dentition phase, four years after RPE.

**Figure 4:**
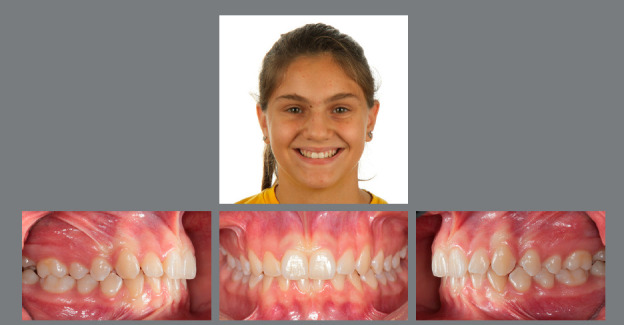
Good stability in the permanent dentition at age 16, eight years after RPE.

The appliance of choice is a tooth-borne Rapid Palatal Expander (RPE) anchored on the second deciduous molars, which is usually activated once a day for four to six weeks, depending on the severity of the transverse discrepancy, and left in place for 9 to 12 months (Figs 1-4). In the absence of additional sagittal or vertical issues, no retention device is necessary. Although Masucci et al.^22^ reported about 30-40% relapse after palatal expansion in the pure deciduous dentition, other research groups described excellent overall long-term stability of very early crossbite correction.^22^ ^-^ ^26^

The authors of the present paper have only very rarely (less than 2%) experienced transverse relapse in their patients, who had to be either retreated by a second RPE or by insertion of a transpalatal bar.

## CLASS III MALOCCLUSION

Since Class III malocclusion tends to worsen during adolescent growth, early interception is recommended, preferably during the deciduous dentition phase, to gain maximum skeletal effect from orthodontic treatment. 

For accurate diagnosis and realistic Class III treatment planning, it is very important to evaluate not only molar and incisor dental relationships, but also to assess any functional Centric Occlusion-Centric Relation (CO-CR) shift on mandibular closure, a cephalometric analysis to determine the amount of underlying sagittal and vertical jaw relationships, and to screen for any very unfavorable genetic predisposition in the family history.^27^ ^,^ ^28^

Especially in cases of hereditary Class III malocclusion, a cephalometric radiograph is mandatory to assess the Wits appraisal, an important diagnostic criterion for successful prognosis of interceptive Class III treatment, and for evaluation of the vertical skeletal dimension. In case of a large Wits value (> - 7mm) associated with a hyperdivergent pattern, the parents should be informed about the looming risk of either a second phase of orthodontic treatment or, in the worst case, of a combined orthodontic-orthognathic approach after the end of the growth period.^29^ ^,^ ^30^

**Figure 5:**
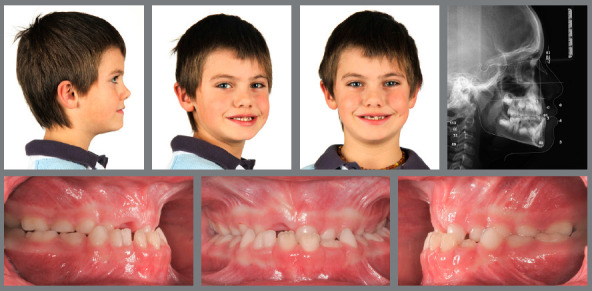
This 6-year-old patient presented a significant low-angle Class III malocclusion, with an anterolateral crossbite.

**Figure 6:**
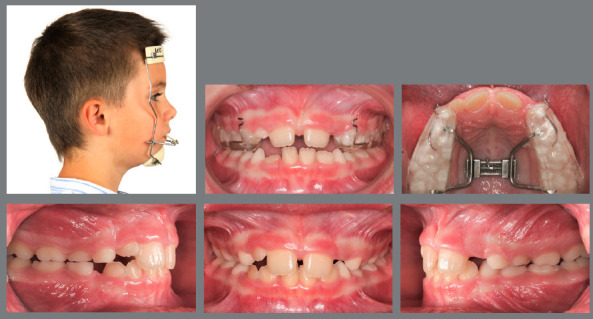
After four weeks of RPE, with one activation per day, a facemask was worn for 12 hours per day, for eight months.

**Figure 7:**
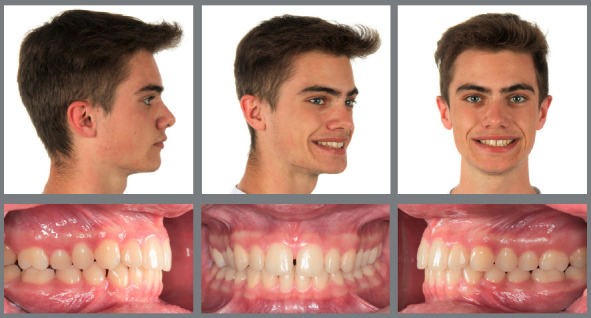
After early interceptive Class III treatment only, no further orthodontic treatment was necessary.

Around 60% of Class III patients^27^ ^,^ ^28^ present a retrusive and constricted maxilla, which means that in 2/3 of these children early interceptive treatment (under age 10) with a facemask attached to a RPE is the method of choice. After the necessary amount of expansion, the maxilla is protracted by a force of 300-600gF per side and with an approximate direction of 30° downward and forward. This approach allows for favorable sutural response of maxillary expansion and protraction, and correction of any CO or CR discrepancies, while the facial profile is improved and self-esteem is enhanced, and works well with mild to moderate Class III malocclusions and with average or reduced vertical proportions (Figs 5-7).^31^ ^-^ ^33^

As the facemask (FM) is mostly worn during night-time only, additional intermaxillary elastics from posterior hooks soldered to the RPE to bonded cleats on the mandibular deciduous canines can help to apply Class III traction almost full-time.

It was reported that early treatment with a facemask appliance has a positive impact on both dental and skeletal parameters, and reduces the need for orthognathic surgery in the future when treatment is performed before the age of 10 years in mild to moderate Class III with a retrusive maxilla and no hyperdivergent facial growth pattern.^34^ ^-^ ^43^ Baccetti et al^44^ ^,^ ^45^ demonstrated that, especially in the pure deciduous dentition at age 5 years, treatment produces more beneficial skeletal effects such as significantly smaller increments in mandibular total length (Co-Pg) compared to more maxillary dentoalveolar protrusion when treatment is performed in the mixed dentition (around 8 years of age).

There is no evidence that adding RPE to a protraction facemask protocol, with the aim to loosen the circummaxillary sutures and to increase forward movement of the maxilla, will enhance maxillary protraction and should therefore only be undertaken in patients with existing transverse maxillary constriction.^46^ ^,^ ^47^

**Figure 8:**
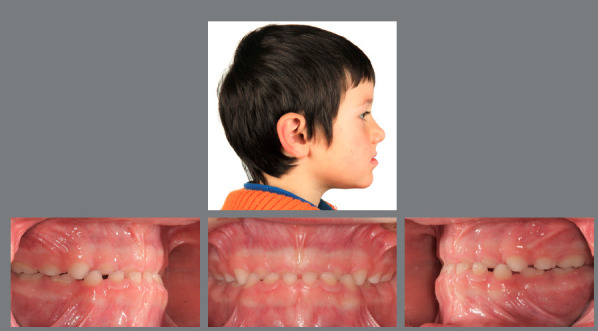
A 5-year-old patient with an anterior crossbite and a deep bite due to hypodivergent Class III facial growth.

**Figure 9:**
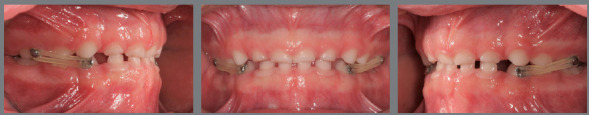
Early treatment was performed with only two Class III elastics (20 hours/day) from the maxillary second deciduous molars to the mandibular canines for six months.

**Figure 10:**
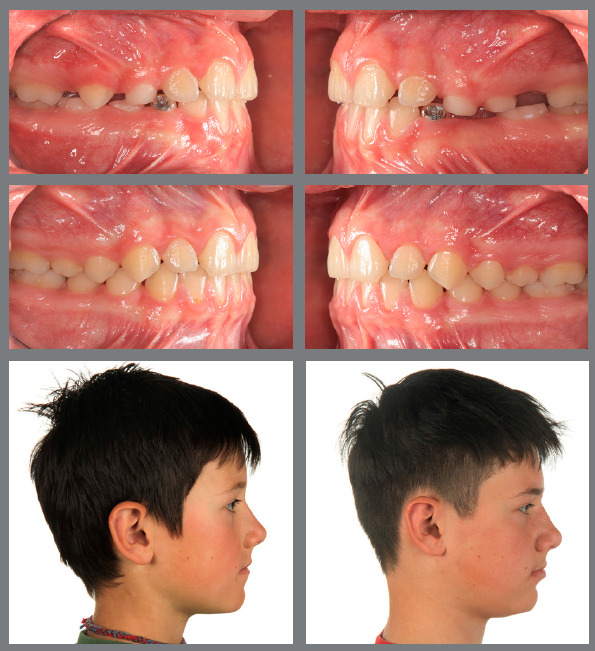
Normalization of the occlusion and significant profile improvement, with good stability of early Class III correction with very simple mechanics at ages 12 and 16 years.

At a very early age (4-5 years), simply bonding cleats or buttons on the maxillary second deciduous molars and on the lower deciduous canines for full-time application of Class III elastics can be an efficient and cheap approach to achieve anterior crossbite correction (Figs 8-10).

With the advent of skeletal anchorage, bone-borne instead of tooth-born maxillary protraction with a facemask has been advocated. Research has shown that tooth-borne facemask protraction leads to more proclination of the maxillary incisors, increased overjet correction, and correction of molar relationship, while bone-anchored facemask protraction achieves greater skeletal effects and reduces undesirable dental compensations, causes less downward movement of point A, less opening of the mandibular plane angle, and more vertical eruption of the maxillary incisors, which is indicated for hyperdivergent Class III patterns.^48^ ^-^ ^53^

To avoid extraoral traction, the use of Class III elastics to a mentoplate has been proposed by Nienkemper et al.^54^ ^,^ ^56^ ^,^ ^57^ and by Sar et al.^55^, and has shown promising results in the short term especially in hyperdivergent patients.

Regardless of the selected mechanical approach for early Class III correction, after this early interceptive phase of treatment, a follow-up lateral cephalogram should be taken 2-4 years after maxillary protraction, to calculate the Growth Treatment Response Vector, as suggested by Ngan et al.^58^ ^,^ ^59^, to determine the individual mandibular growth rate and direction, and to decide whether the malocclusion can be treated by means of orthodontic camouflage or will require future orthognathic surgical correction.

## CLASS II MALOCCLUSION

Although the evidence about the benefit of early treatment for Class II malocclusions is striking, there seems to be a big gap between existing scientific knowledge and its daily clinical application. While numerous well-performed studies have revealed that a two-phase approach is not more effective than a late single approach during the pubertal growth spurt, and can neither significantly reduce the complexity of the second phase -including the necessity of extraction treatments, the percentage of orthognathic surgery or treatment duration of phase II-, it is still advocated by many clinicians.^60^ ^-^ ^64^

Franchi et al.^65^ ^,^ ^66^ clearly evidenced that an early approach of Class II correction is simply ‘overtreatment’, because functional appliance therapy results only in extra mandibular growth if the pubertal stage is incorporated into the treatment plan, which does not occur during the primary and early mixed dentition periods.

The only justification of early intervention of Class II treatment is a mild increased risk of maxillary incisor trauma and psychosocial problems due to bullying.^67^ ^,^ ^68^

However, if Class II malocclusion is associated with either a transverse (lingual or buccal crossbite) or a vertical discrepancy (open bite or deep bite with palatal impingement), early Class II treatment can be advocated. This early approach should be carried out with simple but efficient mechanics, in order not to burn the patient’s compliance and the parent’s economic resources for a potential second phase of treatment during the pubertal growth spurt, which is the ‘gold standard’ for Class II treatment. 

**Figure 11:**
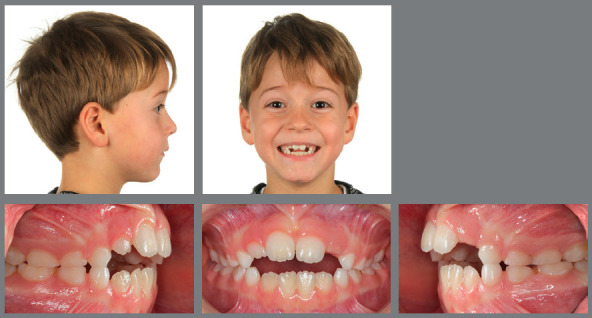
This 7-year-old patient presented a dental open bite with maxillary constriction and mandibular retrusion, leading to occlusal Class II relationships.

**Figure 12:**
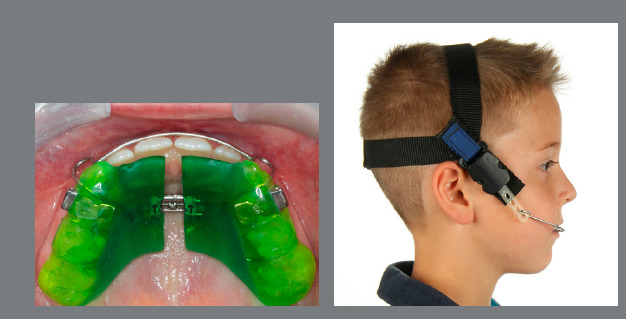
A Joho-plate was worn for 10 months for 14 hours a day.

**Figure 13:**
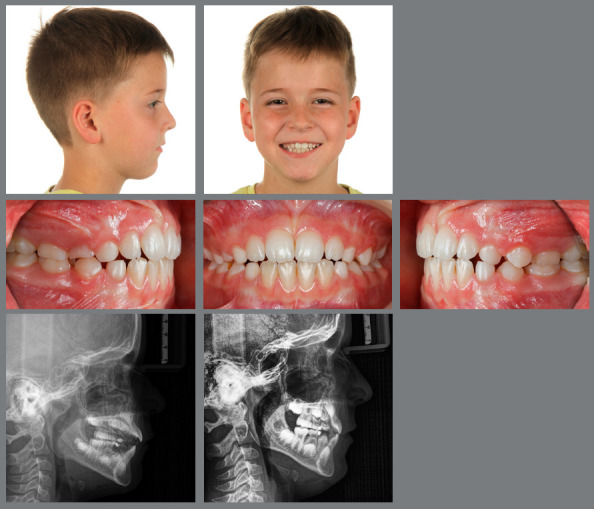
Normal dental and skeletal relationships after early interceptive treatment.

A helpful and efficient appliance for early correction of maxillary constriction, open bite and Class II malocclusion is the removable maxillary Joho-plate, a combination of a removable expansion plate and a highpull-headgear.^69^ With a daily wear time of 12-14 hours, the first active phase of treatment can usually be concluded with 12-15 months of treatment. The plate can then be worn passively during night-time on demand (Figs 11-13).

**Figure 14:**
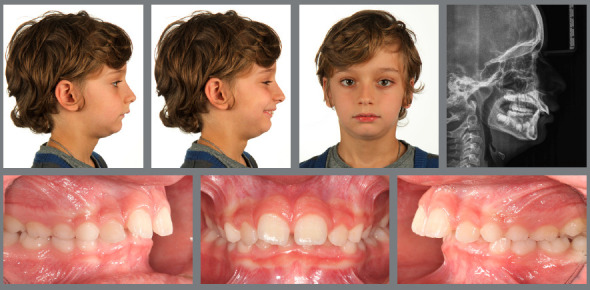
This 8-year-old patient exhibited a full-cusp Class II malocclusion with lip incompetence and palatal impingement, and was bullied at school.

**Figure 15:**
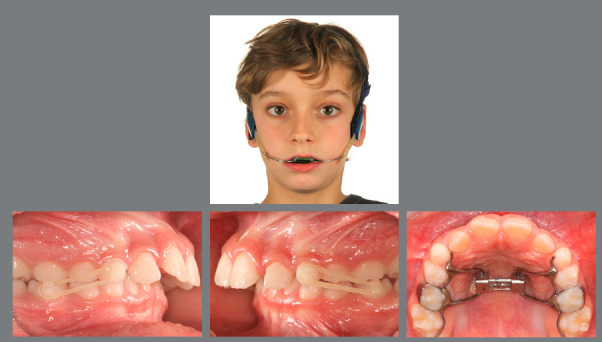
After RPE, a van Beek activator was worn during the night and Class II elastics on bonded resin buttons were applied during the day for 12 months.

**Figure 16:**
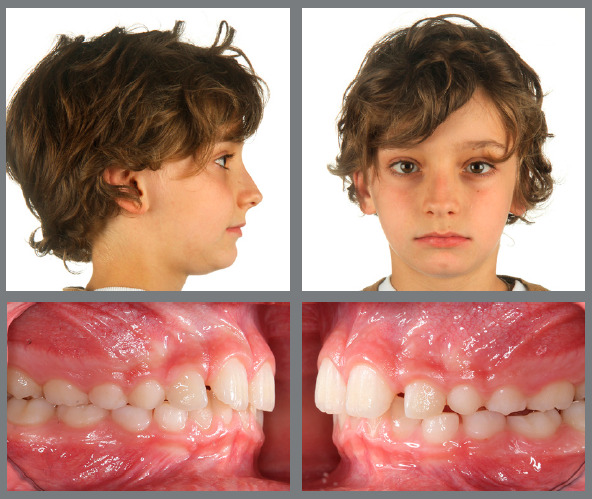
One year of interceptive treatment has corrected the Class II malocclusion and has improved the patient’s profile.

**Figure 17:**
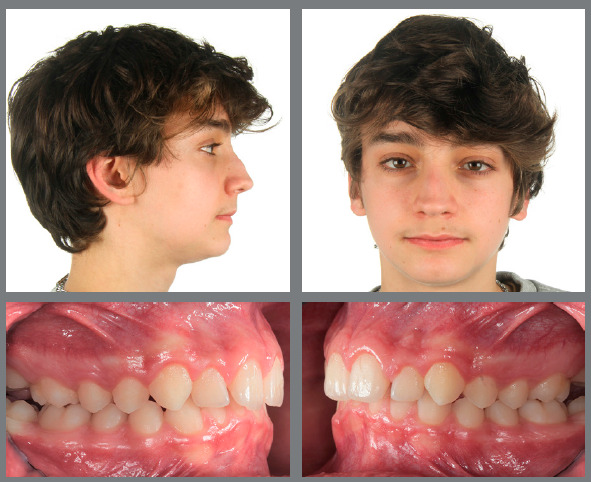
Good maintainability of the early Class II correction can be observed after four years without any further treatment.

In case of a very large overjet with palatal impingement, a first phase of maxillary expansion to accommodate the mandible in advanced position is often necessary and can either be performed with a RPE or a removable expansion plate. Instead of tempting Class II correction with only an activator, which is usually worn only during night-time, light intermaxillary Class II elastics from the mandibular second deciduous molars to the deciduous maxillary canines can be worn over the day and make Class II correction faster and boost the patient’s compliance and satisfaction by reducing the overall treatment time (Figs 14-17).

## OPEN BITE

Successful early orthodontic treatment of open bites depends predominantly on its etiology.^70^ ^-^ ^72^

Successful outcomes can be achieved, if the open bite is mainly due to maxillary transverse constriction and to dental factors caused by either tongue thrust, lip incompetence or sucking habits. In the presence of a mainly dolicofacial growth pattern, early treatment with either rapid (RPE) or slow maxillary expansion (Joho-plate) may not be effective for controlling maxillary downward and forward growth. If such an early approach is undertaken, normal respiratory function must be present. Gracco et al^73^ could evidence that open bites will relapse after orthodontic treatment in the presence of nasal airway breathing problems due to nasal septum deviation, turbinate hypertrophy, and maxillary sinus congestion. They emphasized the necessity of an ENT consultation prior to considering the treatment of anterior open bite.

**Figure 18:**
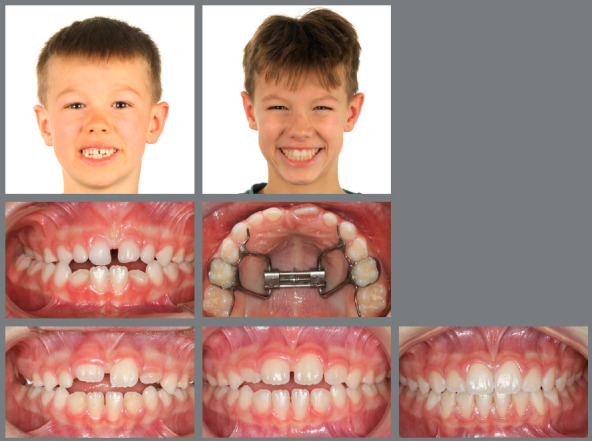
After nine months of RPE for unilateral posterior crossbite correction at age 7, concomitant myofunctional therapy was necessary to close the open bite by elimination of persisting tongue thrust.

Concomitant myofunctional therapy with or without additional tongue repositioning devices, such as spurs or cribs, is advisable to close the anterior open bite (Fig 18).^74^ ^-^ ^77^

It must be acknowledged that stability of open bite treatment is unpredictable irrespective of the treatment modality.^78^ ^-^ ^80^ Hopefully, skeletal anchorage devices will help to increase the amount of orthodontic posterior vertical control and achieve more predictable and more stable results of open bite closure in the future.^81^ ^,^ ^82^

## ARCH LENGTH DISCREPANCY

Substantial hereditary tooth size-arch length discrepancy is a frequent finding already in early childhood, and the crucial question for the orthodontists is whether the appropriate treatment plan is to change the form of the basal bone or the arch form by either expansion, distalization or proclination or to perform a serial extraction treatment approach instead.

In the presence of a lingual crossbite due to maxillary constriction, the first treatment approach will always be maxillary expansion, and the decision to extract or not to extract will be postponed until after the expansion. If no crossbite is present, the decision whether to expand or to extract will depend on the patient’s growth pattern and facial type. Gaining mandibular arch length with a lip bumper may be a feasible option, if the mandibular incisors are in an upright or retruded position, and a favorable Leeway space without any signs of mandibular second molar impaction can be assessed on a panoramic radiograph.

**Figure 19:**
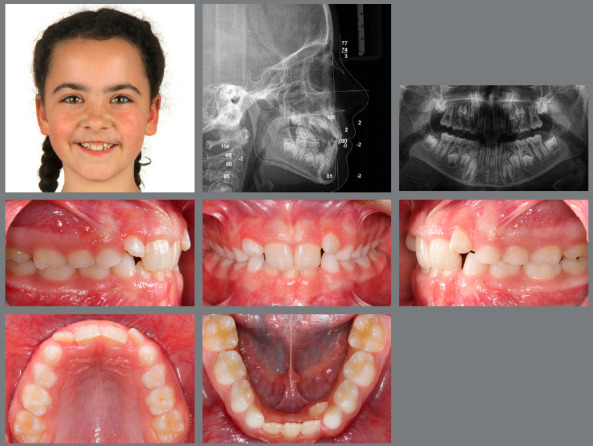
Significant upper and lower arch length discrepancy due to bimaxillary constriction and incisor retrusion.

**Figure 20:**
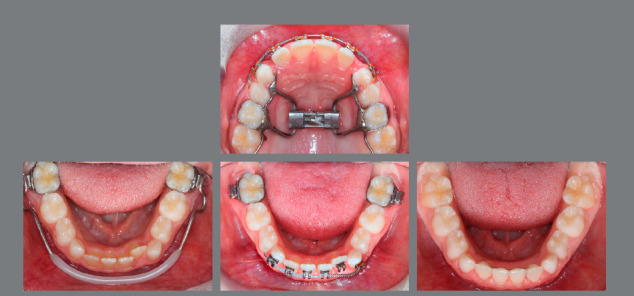
After 9 months of RPE and lip bumper therapy, upper and lower anterior 3-3 brackets were bonded to align the incisors. After 6 months a lower 2-2 lingual retainer was applied.

**Figure 21:**
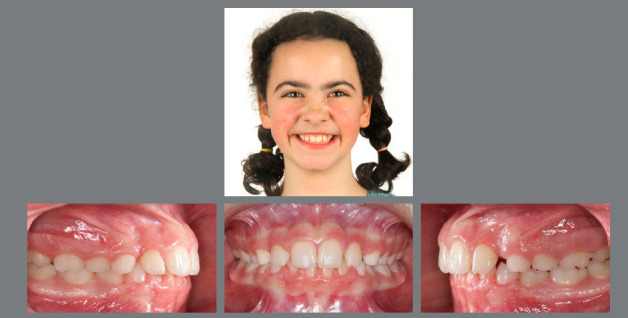
Sufficient arch length has been gained in 15 months of interceptive treatment.

However, the existing sagittal and vertical occlusal and skeletal relationships and the patient’s soft tissue profile have also to be included in the final treatment plan (Figs 19-21).

Apart from an existing significant Class II malocclusion with proclined maxillary incisors, a dental open bite with a hyperdivergent facial pattern and lip incompetence helpful diagnostic criteria that may lead to the decision to extract are:

» Premature exfoliating of one or more lateral incisors with resulting deviation of the dental midline.
» Gingival recession on a prominent lower incisor.
» Splaying out of maxillary or mandibular lateral incisors.
» Ectopic eruption of one or both maxillary first molar(s), with premature exfoliation of the second deciduous molar(s).
» Bimaxillary protrusion.
» Accentuated curves of Spee.
» A vertical palisading of maxillary molars in the tuberosity area.
» Impaction of the mandibular second molars.^83^ ^,^ ^84^

**Figure 22:**
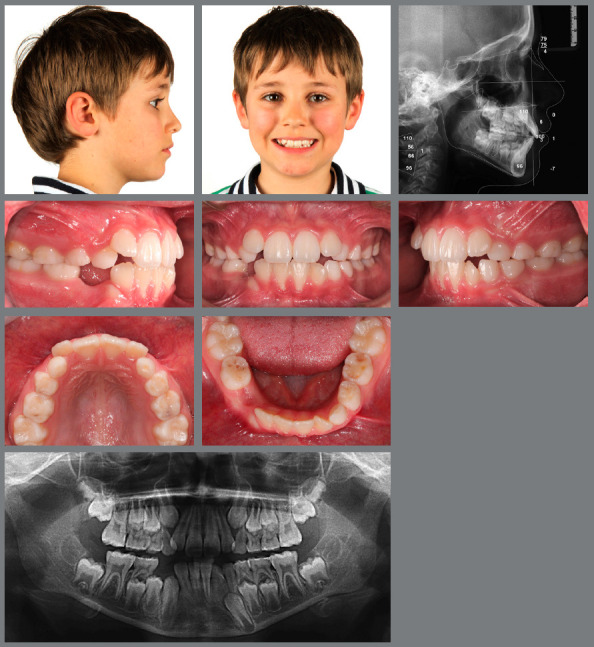
This 9-year-old patient presented a well-balanced profile, a Class I hyperdivergent skeletal pattern and bimaxillary anterior and posterior crowding, with an anterior open bite and a tendency for gingival recession in the mandibular incisor area.

**Figure 23:**
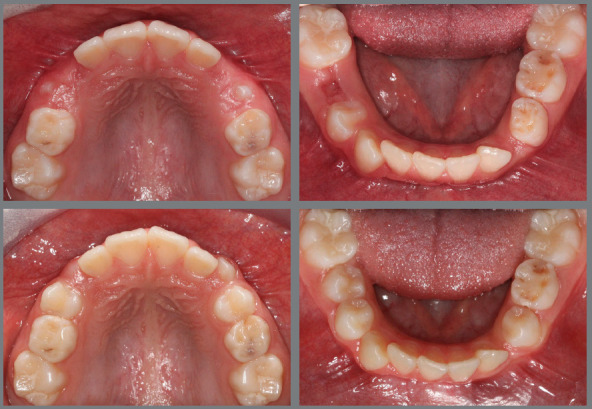
After extraction of all deciduous canines, eruption of the first premolars is monitored. The teeth are then immediately removed and, after complete eruption of the second molars, the necessity for further treatment is re-evaluated.

Once the decision is made that serial extractions are the best treatment option, the extraction of all deciduous canines is prescribed, which will lead to a self-correction of the anterior crowding by tooth migration towards the extraction sites, reduction of bimaxillary protrusion, closure of an existing dental open bite and perhaps even to spontaneous correction of an anterior crossbite (‘driftodontics’) (Figs 22, 23).^85^ ^-^ ^87^

**Figure 24:**
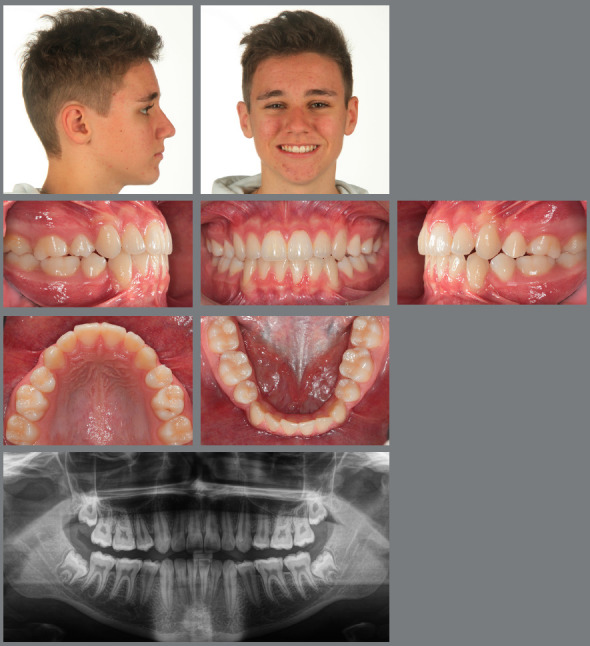
The patient at age 16 without any active orthodontic treatment. All extraction sites have closed spontaneously and a satisfactory Class II occlusion with a normal overbite has developed. The eruption of the third molars needs supervision.

The patient is only seen every six months for monitoring of tooth eruption. Should the mandibular canines tend to erupt prior to the mandibular first premolars, extraction of the mandibular first deciduous molars is advisable to speed up the eruption of the first premolars. Once the maxillary and mandibular premolars have erupted, they will be immediately extracted and monitoring of further tooth eruption is continued until all permanent teeth, including the second molars, have erupted. The beauty of serial extraction treatment is that the natural eruption pathway can be utilized in order to reduce the active treatment time and to keep treatment as comfortable as possible (Fig 24).^88^

Critics of serial extraction treatment often state that early extraction of deciduous canines is a ‘one-way street’ and conditions these patients to pursue the pathway of later premolar extractions. This is not the case, as the final decision whether to extract premolars or to perform any kind of orthodontic arch development is only postponed until eruption of the first premolars and can be critically re-evaluated by the treating orthodontist.

Only once the mandibular second molars have fully erupted, active orthodontic treatment is initiated, which on average takes around 12 to 15 months, depending on the mechanics applied. One of the great advantages of serial extraction treatment is its reduced duration, compared to a two-phase non-extraction or to a late premolar extraction treatment, and may yield more stable long-term results.^89^ ^-^ ^93^

The persisting claims that extraction treatments in general may have a detrimental effect of the patient’s profile or smile width have been sufficiently eradicated in the evidence-based literature which proofs extractions *per se* do not negatively influence facial or smile aesthetics if the indication for extractions is correct and closure of the extraction sites is comprehensively managed. Instead, a categoric rejection of extractions can lead to severe iatrogenic harm of the hard and soft-tissue envelope in terms of provoking dehiscences and gingival recessions, root resorptions, lip procumbency and instability in many patients.^94^ ^-^ ^99^

## CONCLUSION

Early - or even very early - orthodontic treatment with relatively simple and cheap appliances offers an efficient modality for a variety of malocclusions, such as posterior crossbites, mild to moderate Class III problems with maxillary retrusion, dental and mild skeletal open bites and severe Class II malocclusions with associated transverse or vertical alterations. These interceptive treatments should be regarded as ‘short term interventions’ to redirect abnormal growth in 9 to 15 months of treatment, without compromising the child’s compliance. Very often these limited treatments can reduce the length, the discomfort, and the costs of a later second phase of comprehensive treatment with either fixed appliances or clear aligners or, in the best case, avoid the need for a second treatment phase at all. However, in patients with significant tooth size-arch length discrepancies, instead of starting an early phase of expansion treatment with an RPE and a lower arch developer (i.g. lip bumper) the traditional serial extraction should not be completely neglected, as its benefits for certain patients cannot be denied. 
